# New Discoveries in Amyloid‐Related Imaging Abnormalities with Hemorrhage (ARIA‐H) and Anti‐Amyloid Beta Monoclonal Antibodies

**DOI:** 10.1002/alz70855_096374

**Published:** 2025-12-23

**Authors:** Dinghao An, Xinxin Zou, Yun Xu

**Affiliations:** ^1^ Peking Union Medical College, Beijing, Beijing, China; ^2^ Nanjing Drum Tower Hospital, Nanjing, Jiangsu, China; ^3^ Nanjing Drum Tower Hospital Clinical College of Nanjing Medical University, Nanjing, Jiangsu, China

## Abstract

**Background:**

Amyloid‐related Imaging Abnormalities(ARIA) are adverse effects that occur during amyloid beta monoclonal antibody treatment for Alzheimer's disease, including edema‐type ARIA and hemorrhage‐type ARIA(ARIA‐H). Few retrospective analysis have compared ARIA‐H incidence among individual monoclonal antibodies, and a comprehensive comparison is currently lacking. After the approval of these antibodies, research has mainly focused on dosing frequency and drug dosage, leaving it unclear whether ARIA‐H is associated with the specific characteristics of different monoclonal antibodies.

**Method:**

For monoclonal antibodies targeting Aβ clearance, we selected seven: Aducanumab, Bapineuzumab, Crenezumab, Donanemab, Gantenerumab, Lecanemab, and Solanezumab. Our data is derived from systematic reviews and meta‐analyses of Phase III clinical trials. Data from Jeremic D et al.(Ageing Res Rev. 2023 Sep) and Qiao Y et al.(CNS Drugs. 2024 Mar). For the evaluation variables, we selected the following factors: the form of monoclonal antibody binding to Aβ protein, binding affinity, binding epitope, Fc subtype of the monoclonal antibody, and the Aβ clearance rate. ARIA‐H occurrence was used as a categorical variable for differential analysis. We standardized all the data and compared the ARIA‐H occurrence rates for seven mAbs, taking into account other dimensional variables to ensure they were as comparable or similar as possible.

**Result:**

The risk of ARIA‐H, from highest to lowest, is as follows: Donanemab, Aducanumab, Bapineuzumab, Lecanemab, Gantenerumab, Crenezumab, Solanezumab. Besides, ARIA‐H is associated with the characteristics of mAb. (1) More mature Aβ clearance is associated with a higher risk of ARIA‐H.(2) Lower clearance of Aβ oligomers is associated with a higher risk of ARIA‐H.(3) Aβ clearance closer to the *N*‐terminus is associated with a higher risk of ARIA‐H.(4) Monoclonal antibodies with an IgG4 structure are more likely to cause ARIA‐H than those with an IgG1 structure.(5) Faster achievement of Aβ clearance thresholds is associated with a higher risk of ARIA‐H.

**Conclusion:**

This research enhances our understanding of ARIA‐H and may guide future monoclonal antibody drug development, which may improve the cognition and overall prognosis of Alzheimer's disease patients.

